# Influence of differential leadership behavior on employees’ deviant innovation: Based on dual perspectives of insider and outsider subordinates

**DOI:** 10.3389/fpsyg.2022.945598

**Published:** 2022-08-23

**Authors:** Jie Lu, Linrong Zhang, Mengyun Wu, Muhammad Imran, Qi He, Yun Zhao

**Affiliations:** ^1^School of Management, Jiangsu University, Zhenjiang, China; ^2^School of Finance & Economics, Jiangsu University, Zhenjiang, China; ^3^Information Research Institute, Qilu University of Technology (Shandong Academy of Sciences), Jinan, China; ^4^Department of Economics and Management, Yuncheng University, Yuncheng, China

**Keywords:** differential leadership, deviant innovation behavior, insider subordinates, outsider subordinates, Game Theory

## Abstract

Differential leadership as a localized leadership style gradually developed on the basis of the Pattern of Differential Sequence. It plays a dual role in stimulating “insider subordinates” and “outsider subordinates” through the dynamic transformation of the roles. Using the process of game reasoning, the study identifies the differing principles used by insider subordinates and outsider subordinates in implementing deviant innovative behaviors. The simulation graph presents the perceived benefits of employees performing or not performing deviant innovative behaviors as clues during the reasoning process, and implements deviant innovative behaviors for the high risk-taking trait of insider subordinates and the high internal control personality of outsider subordinates who implement deviant innovation. The theoretical derivation of behavior provides relevant references, and provides counter measures for effectively promoting employees’ deviant innovative behavior in the context of differential leadership.

## Introduction

Differential leadership refers to the differing leadership behaviors of leaders toward their subordinates under the conditions of personal rule, it is a localized leadership style gradually developed on the basis of the Differential Sequence Pattern which stems from the traditional Chinese social structure, and is a social relationship formed by self-focus and kinship as a bond. As we know, differential leadership is a kind of localized leadership style, and it achieves procedural equality through the dynamic transformation of the roles of *subordinates inside the circle* and *subordinates outside the circle*, which is in line with employees’ perception of differential treatment. The psychological expectations of the flow mechanism can play a dual role in inspiring *subordinates inside the circle* and *subordinates outside the circle* (Lin and Cheng, 2017). In some sense, differential leadership encourages *subordinates outside the circle* to work hard to improve performance, and to seek opportunities for interaction and transfer to the core of the organizational structure. Thus, firstly, this study provides an integrated discussion on the choice of pro-organizational strategies of employees under the situation of differential leadership, which enlightens this study to incorporate both the roles of *inside subordinates* and *outside subordinates* into a unified framework, so as to finally achieve leadership effectiveness and organizational goals. Secondly, the framework innovatively uses the game simulation model to demonstrate the different principles of deviant innovation behavior of *inside subordinates* and *outside subordinates*, and provides countermeasures for effectively promoting employees’ deviant innovation behavior in the context of differential leadership.

On the one hand, when the *insider subordinates* cannot meet their leader’s role requirements or meet their leader’s expectations, the leader may also gradually weaken or dissolve the business relationship with *insider subordinates*, which results in the emergence of conversion of the latter from being *subordinates inside the circle* to *subordinates outside the circle* (Liu, 2014; Wu et al., 2018b). Therefore, differential leadership can motivate *insider subordinates* to improve their work ability, improve work efficiency, and fully explore their own advantages in order to avoid being replaced by other employees.

On the other hand, when the *subordinates outside the circle* meet the basic conditions of the category of *subordinates inside the circle* by cooperating with management requirements, showing positive actions, and establishing relationship carriers, it is possible for the leader to transfer such employees from the group of *outside subordinates* to that of insiders (Gao and Wang, 2013). Therefore, differential leadership can encourage *outside subordinates* to work hard to improve their performance evaluation, and strive for opportunities to achieve interactive transformation and then move to the core of the organizational structure.

Following the formation of an appropriate quantitative approach for differential leadership, empirical studies have confirmed that *subordinates within the circle* enhance role stability, and *subordinates outside the circle* have a positive impact on work performance by changing alienation patterns (Lin and Cheng, 2017). This also provides an integrated discussion of the pro-organizational strategy choice of employees in the context of differential leadership, which leads to this study incorporating both the roles of *subordinates inside the circle* and *subordinates outside the circle* into the mixed research framework of differential leadership theory. The study also uses Game Theory, a mathematical theory and method for studying phenomena of a struggle of a competitive nature and the interaction of formulaic incentive structures, to apply game reasoning process to find out the different principles of deviant innovation behavior between *inside subordinates* and outside *subordinates.*

Hence the mutual transformation between *inside subordinates* and *outside subordinates* does not mean the transition is a rule from *subordinates in the circle* to *subordinates out of the circle* and the inversion changes of each are completely equivalent. In fact, it is easy to understand that the essence of the process of classification conversion between *subordinates inside the circle* and *subordinates outside the circle* is more due to the adjustment of leaders’ views on employees. Most leaders aim to use the basis of *subordinates inside the circle* to expand their control and acquire more resources in order to strengthen their influence (Shen, 2012; Wu et al., 2018a). The important characteristics formed within the organization are the immobilization trend of *subordinates inside the circle* and the changing trend of *subordinates outside the circle*. It can be seen that the change from “outside subordinate” to “inner subordinate” may have a higher probability, because the people outside the circle are more motivated and inclined to gain the approval of their leaders and move closer to the leader’s circle.

Therefore, the purpose of differential leadership is to integrate the *subordinates inside the circle* and *subordinates outside the circle* into a unified framework through management measures directed at differentiated treatment, and thus ultimately achieve leadership effectiveness and meet organizational goals. The contribution of the study is: firstly, analyzing the adaptability of employees’ deviant innovative behaviors from the perspective of cultural psychology has become an urgent need in the context of China’s local management. This study provides a theoretical reference for demonstrating whether leaders’ management methods and behavioral models under a specific cultural premise can stimulate employees’ work behaviors and their effectiveness. Secondly, some studies confuse the inherent differences between the deviant innovative behaviors of “inner subordinates” and “outside subordinates” caused by differential leadership. This study selects risk-taking traits and internal control personality as triggering conditions, avoiding the ambiguity in understanding of the mechanism between differential leadership and employee deviant innovative behavior. Thirdly, existed research mostly relied on mathematical statistics to verify the mechanism and boundary conditions of differential leadership affecting employees’ deviant innovation behavior. This study uses evolutionary game and simulation analysis methods to systematically infer the behavioral laws of micro-individuals and their evolutionary processes, and examines the specific details of the impact of differential leadership on employees’ deviant innovative behavior, so as to fully grasp the psychological state and behavior of employees in the context of differential leadership.

## Game reasoning on influence of differential leadership on employees’ deviant innovation behavior

Affected by the long-term influence of traditional Chinese circle culture, differential leaders will divide employees into *subordinates inside the circle* and *subordinates outside the circle* within their Chinese organization (Li, 2019). In response to the treatment methods cited above, employees will form a self-role perception of *insider subordinates* or *outsider subordinates*, and form different inner states based on their respective roles, and continue to promote rational behavior choices. For a long time innovative behavior has been widely regarded as an inexhaustible driving force for organizational development, but enterprises have gradually discovered that in practice employee innovation behavior has begun to be tinged with deviance, and innovation activities and workplace deviance are potentially closely and interestingly related (Criscuolo et al., 2015; Wu et al., 2020).

To give examples of such deviance and innovation, in the long history of enterprise development, the advent of the Sogou browser, the birth of 3M transparent tape, and the development of HP’s new monitor all originated in deviant innovation by employees and all had a subversive impact on their enterprises. Deviant innovative behaviors seem to violate organizational norms, but if scientific guidance is used to effectively enhance its positive effects and intervene in its negative effects, it can help enterprises maximize the effectiveness of resources and effectively break innovation bottlenecks. It is an effective means of organizational innovation in the new era (Gao et al., 2020).

Although the inner psychological process experienced by *subordinates inside the circle* and *subordinates outside the circle* is different, this also implies the possibility of differential leadership to create deviant innovative behaviors of employees. Because of the leadership’s differential leadership, the subordinates of the insiders will dare to carry out various innovative practice activities, especially when their innovative ideas are immature, but, due to the low cost of trial and error, the insiders dare not do so through the leader’s consent process in order to carry out their deviant innovation activities. Conversely, for the subordinates outside the circle, they will feel a sense of rejection because of the leader’s partiality, and they are eager to gain recognition and integrate into the group. This study seeks to build a differential organizational structure with leaders as the center and to spread outward, and it constitutes four types of employees: *insider subordinates* who perform deviant innovation behaviors, *insider subordinates* who do not perform deviant innovation behaviors, *outside subordinates* who practice deviant innovative behaviors and *outside subordinates* who do not implement deviant innovative behaviors. At the same time, the risk-taking characteristics of *subordinates of inner circle* and the internal control personality of subordinates of outside circle are taken into account. Based on the consideration of when facing with the current advantageous situation whether inner subordinates are willing to “content with the status quo,” this study chooses the risk-taking trait as the boundary condition for whether inner subordinates will make aggressive behavior. And also, this study selects the internal control personality as the boundary condition for whether outside subordinates will take breakthrough behaviors based on the belief that “Man by his efforts can conquer nature.” Analyzing the risk characteristics of subordinates inside the circle and the internal control personality of subordinates outside the circle can better help identifying the possibility of employees’ deviant innovative behaviors, and help employees gain a better sense of corporate identity on the premise of deviant but not out of control.

### The first stage

In an ideal organizational environment, members of different organizations can directly or indirectly share information through the interaction of network nodes, and ultimately can fully grasp the comprehensive connotation of information in the workplace. However, the interaction characteristics of organizational members in a real organizational situation are that they maintain uniform contact or random contact with other organizational members around them. They cannot achieve a fully coupled state, so that they are only partially rational (Zhao et al., 2019; Wu et al., 2021). In the face of the objective background of information asymmetry, organizational members can only measure their own profit index within the organizational network by comparison with other members on relevant network nodes.

The game participants proposed in this study include *insider subordinates* and *outside subordinates*. This assumes that there are two behaviors in the organization at the present time: “implementing deviant innovative behaviors” and “not implementing deviant innovative behaviors.” That is, if both employees participating in the game adopt the strategic choice of “implementing deviant innovative behaviors,” the actual benefits obtained by both parties are *P*; if the two employees participating in the game choose different strategies, the actual benefit of the employee who adopts the strategy of “implementing deviant innovative behaviors” will be *S*, and the actual benefit of employees who choose the strategy of “refusing implement deviant innovative behaviors” will be *T*; if both employees participating in the game take the strategic choice of “not implementing deviant innovative behaviors,” then the actual benefit obtained by both parties is *R*. From this, we can devise the actual income game matrix obtained by the employees in adopting the strategy of “implementing deviant innovative behaviors” or “not implementing deviant innovative behaviors,” as shown in Table 1.

**TABLE 1:** Game matrix of real benefit.

	Outsiders carry out deviant innovative behaviors	Outsiders do not carry out deviant innovative behaviors
Insiders carry out deviant innovative behaviors	*(P, P)*	*(S, T)*
Insiders do not implement deviant innovative behaviors	*(T, S)*	*(R, R)*

Therefore, this study infers that employee *i* will accumulate a comprehensive actual income $f_{1}^{i}\left(m\right)$ after playing a total of m games with employee *j* on the relevant network nodes. The calculation method is shown in Equation (1):


(1) $$\begin{array}{c} {\text{f}}_{1}^{\text{i}}\left(m\right)=\sum_{\text{j}\in \partial \text{i}}[\frac{1}{4}\left(1-{\text{s}}_{\text{i}}\right)\left(1-{\text{s}}_{\text{j}}\right)\text{P}+\frac{1}{4}\left(1-{\text{s}}_{\text{i}}\right)\left(1+{\text{s}}_{\text{j}}\right)\text{S}\\ \;+\frac{1}{4}\left(1+{\text{s}}_{\text{i}}\right)\left(1-{\text{s}}_{\text{j}}\right)\text{T}+\frac{1}{4}\left(1+{\text{s}}_{\text{i}}\right)\left(1+{\text{s}}_{\text{j}}\right)\text{R}] \end{array}$$

where *s*_*i*_ = −1, which means that employee *i* adopts the strategy choice of implementing deviant innovative behaviors; *s*_*i*_ = 1 means that employee *i* adopts the strategy choice of not implementing deviant innovation behaviors; and δ_*i*_ means the set of relevant network nodes of employee *i*.

Following the comprehensive actual benefit function of Equation (1) and the corresponding matching angle, the comprehensive actual benefit $f_{1}^{C}\left(m\right)$ obtained by employee *i* by performing deviant innovative behaviors and the comprehensive actual benefit obtained by employee *i* by not performing deviant innovative behaviors can be obtained, respectively. The income $f_{1}^{N}\left(m\right)$, the specific parameter equation, is obtained as follows:


(2) $${\text{f}}_{1}^{\text{C}}\left(m\right)=\sum_{\text{j}\in \partial \text{i}}\left[\frac{1}{2}\left(1-{\text{s}}_{\text{j}}\right)\text{P}+\frac{1}{2}\left(1+{\text{s}}_{\text{j}}\right)\text{S}\right]$$

(3) $${\text{f}}_{1}^{\text{N}}\left(m\right)=\sum_{\text{j}\in \partial \text{i}}\left[\frac{1}{2}\left(1-{\text{s}}_{\text{j}}\right)\text{T}+\frac{1}{2}\left(1+{\text{s}}_{\text{j}}\right)\text{R}\right]$$

According to the principle of differentiation between *insider subordinates* and *outsider subordinates* of differential leadership, the difference in attitude toward *subordinates inside the circle* and disliked treatment of *subordinates outside the circle* will lead to differences in benefits between those two. This will inevitably lead to the comprehensive actual benefits of *subordinates inside the circle* that are obviously different from those of subordinates of *subordinates outside the circle*, and the comprehensive actual income of *subordinates outside the circle*. Therefore, there are four different levels of comprehensive actual income, such as *subordinates inside the circle* who implement deviant innovative behaviors, *subordinates inside the circle* who do not implement deviant innovative behaviors, *subordinates outside the circle* who implement deviant innovative behaviors, and *subordinates outside the circle* who do not implement deviant innovative behaviors. The specific parameter settings are shown in $f_{1}^{C}\left(m\right)$$f_{1}^{IN}\left(m\right)$$f_{1}^{OC}\left(m\right)$$f_{1}^{ON}\left(m\right)$:


(4) $${\text{f}}_{1}^{\text{IC}}\left(m\right)=\sum_{\text{j}\in \partial \text{i}}\left[\frac{1}{2}\left(1-{\text{s}}_{\text{j}}\right)\text{P}+\frac{1}{2}\left(1+{\text{s}}_{\text{j}}\right)\text{S}\right]+\alpha \text{U}$$

(5) $${\text{f}}_{1}^{\text{IN}}\left(m\right)=\sum_{\text{j}\in \partial \text{i}}\left[\frac{1}{2}\left(1-{\text{s}}_{\text{j}}\right)\text{T}+\frac{1}{2}\left(1+{\text{s}}_{\text{j}}\right)\text{R}\right]+\alpha \text{U}$$

(6) $${\text{f}}_{1}^{\text{OC}}\left(m\right)=\sum_{\text{j}\in \partial \text{i}}\left[\frac{1}{2}\left(1-{\text{s}}_{\text{j}}\right)\text{P}+\frac{1}{2}\left(1+{\text{s}}_{\text{j}}\right)\text{S}\right]+\alpha \text{U}$$

(7) $${\text{f}}_{1}^{\text{ON}}\left(m\right)=\sum_{\text{j}\in \partial \text{i}}\left[\frac{1}{2}\left(1-{\text{s}}_{\text{j}}\right)\text{T}+\frac{1}{2}\left(1+{\text{s}}_{\text{j}}\right)\text{R}\right]+\alpha \text{U}$$

As stated in the above equations, Equation (4) represents the comprehensive actual income $f_{1}^{C}\left(m\right)$ that can be obtained by “insider subordinates” who carry out deviant innovative behaviors. Equation (5) represents the comprehensive actual income $f_{1}^{IN}\left(m\right)$ that can be obtained by *insider subordinates* who do not carry out deviant innovative behaviors. Equation (6) represents the comprehensive actual income $f_{1}^{OC}\left(m\right)$ that can be obtained by *outside subordinates* who carry out deviant innovative behaviors. Equation (7) represents the comprehensive income $f_{1}^{ON}\left(m\right)$that *outside subordinates* can obtain without deviant innovative behaviors. The conclusions are ${\text{f}}_{1}^{\text{IC}}\left(m\right)={\text{f}}_{1}^{\text{OC}}\left(m\right)$ and ${\text{f}}_{1}^{\text{IN}}\left(m\right)={\text{f}}_{1}^{\text{ON}}\left(m\right)$.

In addition, the differential ordering style of differential leadership will form a non-equivalent comprehensive income between *subordinates inside the circle* and *subordinates outside the circle*. That means that it will generate a moderate comprehensive income that promotes growth through partiality treatment, and through preferential treatment it produces a moderately downward comprehensive income, thus creating an absolute gap in the comprehensive income of “subordinates inside the circle” and “subordinates outside the circle,” which can be represented by *U*, and where α is used to represent the difference order. The difference between *insider subordinates* and *outsider subordinates* will be affected by styles of leadership.

### The second stage

Deviant innovation behavior is composed of two heterogeneous connotations of *deviance* and *innovation*. Ignoring or violating the core factors of the organizational system can easily make leaders resentful, but undoing the implementation of innovative ideas and passively maintaining the current situation is also difficult if the aim it to engage the goodwill of leaders (Wang and Wang, 2020). Regardless of whether they are *insider subordinates* or *outsiders*, even if they intend to improve the overall innovation performance of the organization and self-innovation benefits through deviant innovative behaviors, or they have no intention of implementing deviant innovative behaviors and adhere to their own responsibilities, there is a certain difficulty in the actual benefits that can be obtained from deviant innovative behaviors and non-implemented deviant innovative behaviors. So both *subordinates inside the circle* and *subordinates outside the circle* are the path choices of implementing or not implementing deviant innovative behaviors, and determining one’s own behavior choices based on the perceived benefits of implementing or not implementing deviant innovative behaviors is shown in the parametric equations obtained:


(8) f2i(m)=f1i(m)*exp[-(-lnWsi)]γ

In the above equation, exp[-(-lnWsi)]γ represents the trade-off factor between the actual benefit and the perceived benefit of the employee, where ${\text{W}}_{{\text{s}}_{\text{i}}}=\frac{{\text{f}}_{1}^{\text{i}}\left(m\right)*\left({\text{s}}_{\text{i}},-1\right)+{\text{f}}_{1}^{\text{i}}\left(m\right)*\left({\text{s}}_{\text{i}},1\right)}{\sum_{\text{j}=1}^{\partial \text{i}}{\text{f}}_{1}^{\text{j}}\left(m\right)},\left(x,y\right)=\left\{ \begin{array}{c} 1,x=\text{y}\\ 0,x\neq \text{y} \end{array}\right.,\gamma \in \left[0,1\right]$ represents the rational perception coefficient of employees.

Following the perceived benefit function of Equation (8) and the corresponding matching angle, the perceived benefit ${\text{f}}_{2}^{\text{C}}\left(m\right)$ of employees who perform deviant innovative behaviors and the perceived benefits ${\text{f}}_{2}^{\text{N}}\left(m\right)$ of employees who do not perform deviant innovative behaviors can be obtained. The specific parameter equations are as follows:


(9) f2C(m)=f1C(m)*exp[-(-lnWsC)]γ

(10) f2N(m)=f1N(m)*exp[-(-lnWsN)]γ

In addition, this study involves the implementation of deviant innovative behaviors by *insider subordinates*, the implementation and non-implementation of deviant innovative behaviors by *insider subordinates*, and the implementation and non-implementation of deviant innovative behaviors by *outside subordinates*. Thus the perceived benefit function of two different behavioral strategies for choosing to implement or not to implement deviant innovative behaviors for two different types of employees, namely *insider subordinates* and *outside subordinates*, should be set as follows:


(11) f2IC(m)=f1IC(m)*exp[-(-lnWsIC)]γ

(12) f2IN(m)=f1IN(m)*exp[-(-lnWsIN)]γ

(13) f2OC(m)=f1OC(m)*exp[-(-lnWsOC)]γ

(14) f2ON(m)=f1ON(m)*exp[-(-lnWsON)]γ

Therefore, this study sets corresponding parameters for subsequent reasoning, as shown in the above four equations. In these, ${\text{f}}_{2}^{\text{IC}}\left(m\right)$ refers to the perceived benefits of *insider subordinates* for implementing deviant innovative behaviors, ${\text{f}}_{2}^{\text{IN}}\left(m\right)$ refers to the perceived benefits of *insider subordinates* for not implementing deviant innovative behaviors, ${\text{f}}_{2}^{\text{OC}}\left(m\right)$ refers to the perceived benefit of *outside subordinates* for implementing deviant innovative behaviors, and ${\text{f}}_{2}^{\text{ON}}\left(m\right)$ refers to the perceived benefit of *outside subordinates* for not implementing deviant innovative behaviors.

Due to the actual benefit distance caused by the difference in the attitude of differential leaders toward *insider subordinates* and *outside subordinates*, both parties can estimate the benefits of implementing deviant innovative behaviors or not implementing deviant innovative behaviors. At the time, it is recognized that the perceived benefit caused by the deviant innovative behaviors of *subordinates inside the circle* must be higher than the perceived benefits of *subordinates outside the circle* caused by deviant innovative behaviors, that is, ${\text{f}}_{2}^{\text{IC}}\left(m\right)={\text{f}}_{2}^{\text{OC}}\left(m\right)$; and the perceived benefit caused by *subordinates inside the circle* not performing deviant innovative behaviors must be higher than the perceived benefits caused by *subordinates outside the circle* not performing deviant innovative behaviors, that is, ${\text{f}}_{2}^{\text{IN}}\left(m\right)={\text{f}}_{2}^{\text{ON}}\left(m\right)$.

Through the above analytical process, the study found that, under the same behavioral premise, the perceived benefits of *subordinates inside the circle* are higher than those of *outsiders*.

### The third stage

#### Risk-taking trait of insider subordinates

*Insider subordinates* are influenced by risk-taking traits and choose whether to implement deviant innovative behaviors. *Insider subordinates* with high risk-taking traits will choose to implement deviant innovative behaviors because they value the possibility of benefit. Low risk-taking traits of *insider subordinates* will choose not to implement deviant innovative behaviors out of fear of the possibility of loss (Venkatraman, 1991). Therefore, the perceived benefits of *insider subordinates* who carry out deviant innovative behaviors with high risk-taking traits are usually higher than the perceived benefits generated by *insider subordinates* who do not perform deviant innovative behaviors: it is equal to ${\text{f}}_{3}^{\text{ICH}}\left(\text{m}\right)>{\text{f}}_{3}^{\text{INH}}\left(\text{m}\right).$ Conversely, the perceived benefits of the low-risk-type *insider subordinates* who do not carry out deviant innovative behaviors are usually higher than the perceived benefits of *insider subordinates* who carry out deviant innovative behaviors, equal to ${\text{f}}_{3}^{\text{INL}}\left(\text{m}\right)>{\text{f}}_{3}^{\text{ICL}}\left(\text{m}\right)$. The specific parameter settings are shown in the following equations:


(15) $${\text{f}}_{3}^{\text{ICH}}\left(\text{m}\right)={\text{f}}_{2}^{\text{IC}}\left(\text{m}\right)+\varphi \text{D}$$

(16) $${\text{f}}_{3}^{\text{ICL}}\left(\text{m}\right)={\text{f}}_{2}^{\text{IC}}\left(\text{m}\right)-\varphi \text{D}$$

(17) $${\text{f}}_{3}^{\text{INH}}\left(\text{m}\right)={\text{f}}_{2}^{\text{IN}}\left(\text{m}\right)-\varphi \text{D}$$

(18) $${\text{f}}_{3}^{\text{INL}}\left(\text{m}\right)={\text{f}}_{2}^{\text{IN}}\left(\text{m}\right)+\varphi \text{D}$$

As stated in the above equations, in this study, Equation (15) and Equation (16) represent the perceived benefits that can be obtained by the high and low risk-taking traits of *insider subordinates* who carry out deviant innovative behaviors; Equation (17) and Equation (18) represent the perceived benefits that can be obtained by the high and low risk-taking traits of *insider subordinates* who do not carry out deviant innovative behaviors. In addition, the different levels of risk-taking traits of *insider subordinates* will lead to unequal perceived benefits between performing deviant innovative behaviors and not doing so. This is equivalent to being under the condition that *insider subordinates* implement deviant innovative behaviors, that through high risk-taking traits, they produce moderately increasing perceived benefits, and through low risk-taking traits, they produce moderately decreasing perceived benefits. Under the condition of no deviant innovation behavior, the perceived benefit of moderately pushing down is through high risk-taking traits, and the perceived benefit of moderately increasing is through low risk-taking traits. Therefore, the risk-taking trait will separate the subjective gap between the perceived benefits of *insider subordinates* who perform deviant innovative behaviors and those who do not perform deviant innovative behaviors, which can be represented by *D*; φ is used to represent the level difference coefficient of the risk-taking trait of *insider subordinates*.

#### Internal control personality of subordinates outside of the circle

Influenced by the internal control personality, *subordinates outside the circle* choose whether to implement deviant innovative behaviors; *outside subordinates* with high internal control personality will choose to implement deviant innovative behaviors because they detect the possibility of success, while *outside subordinates* with low internal control personality will choose not to implement deviant innovative behaviors because they deny the possibility of success (Joe, 1971). Therefore, the perceived benefits of *outside subordinates* who carry out deviant innovative behaviors with high internal control personality are usually higher than the perceived benefits generated by *outside subordinates* who do not perform deviant innovative behaviors: this is equal to ${\text{f}}_{3}^{\text{OCH}}\left(\text{m}\right)>{\text{f}}_{3}^{\text{ONH}}\left(\text{m}\right).$ Conversely, the perceived benefits of *outside subordinates* who do not carry out deviant innovative behaviors with low internal control personality are usually higher than the perceived benefits generated by *outside subordinates* who carry out deviant innovative behaviors: this is equal to ${\text{f}}_{3}^{\text{ONL}}\left(\text{m}\right)>{\text{f}}_{3}^{\text{OCL}}\left(\text{m}\right)$. The specific parameter settings are as follows:


(19) $${\text{f}}_{3}^{\text{OCH}}\left(\text{m}\right)={\text{f}}_{2}^{\text{OC}}\left(\text{m}\right)+\delta \text{A}$$

(20) $${\text{f}}_{3}^{\text{OCL}}\left(\text{m}\right)={\text{f}}_{2}^{\text{OC}}\left(\text{m}\right)$$

(21) $${\text{f}}_{3}^{\text{ONH}}\left(\text{m}\right)={\text{f}}_{2}^{\text{ON}}\left(\text{m}\right)-\delta \text{A}$$

(22) $${\text{f}}_{3}^{\text{ONL}}\left(\text{m}\right)={\text{f}}_{2}^{\text{ON}}\left(\text{m}\right)$$

As stated in the above equations, in this study, Equation (19) and Equation (20) are used to represent the perceived benefits that can be obtained by *outside subordinates* with high and low internal control personality by implementing deviant innovative behaviors; and Equation (21) and Equation (22) represent the perceived benefits that can be obtained by high and low internal control personality of *outside subordinates* who do not carry out deviant innovative behaviors. In addition, the different internal-controlling personality levels of *subordinates outside the circle* will lead to unequal perceived benefits between the implementation of deviant innovative behaviors and the non-deviant innovative behaviors. This is equivalent to being under the condition that *outside subordinates* carry out deviant innovative behaviors, and a medium-to-growth perceived benefit can be generated through high internal control personality, while *outside subordinates* with low internal control personality stubbornly reject the meaning of behavior, so that this will not bring perceived gains and losses.

Under the condition that *outside subordinates* do not perform deviant innovative behaviors, a moderately downward perceived benefit is produced through high internal control personality, while *outside subordinates* with low internal control personality originally veto the meaning of behavior, so that they will not increase or decrease in perceived benefits. Therefore, the internal control personality will separate the subjective gap between the perceived benefits of *outside subordinates* who implement deviant innovative behaviors and those who do not implement deviant innovative behaviors, which can be represented by A, where δ is used to represent the horizontal difference coefficient of the internal control personality of *subordinates outside the circle*.

### The fourth stage

In an organizational setting, any employee is likely to weigh the possible benefits in relation to their own behavioral strategies, and make embedded comparisons based on information which was collected about the relevant organizational members. Once they perceive that their own profit prospects in the current situation are poor compared to the expected gains and losses of the other situation, they will try to learn from the experience of others to make behavioral adjustments in order to have the opportunity to gain more (Yan and Ji, 2022). In other words, at some point after the end of the previous game round, employee *i* will measure the perceived profit of the next game, and then randomly select employee *j* on the relevant nodes in the organization network and make profit estimates from his standpoint, which then leads the subsequent decision to continue or change their action strategy according to the comparison results of the two perceived benefits. Therefore, the Fermi criterion is applied in this study to imitate the probability of employee *i* selecting employee *j*’s action strategy on relevant network nodes to conduct behavior update, as shown in Equation (23):


(23) $$\text{p}\left({\text{s}}_{\text{i}}\leftarrow {\text{s}}_{\text{j}}\right)=\frac{1}{1+\text{exp}\left[-\frac{{\text{f}}_{3}^{\text{j}}\left(\text{m}\right)-{\text{f}}_{3}^{\text{i}}\left(\text{m}\right)}{\kappa }\right]}$$

Here ${\text{f}}_{3}^{\text{i}}\left(\text{m}\right){\text{ and f}}_{3}^{\text{j}}\left(\text{m}\right)$, respectively, represent the perceived benefits that employee *i* can generate based on his own behavioral strategy, and the perceived benefits that employee *i* can generate based on employee *j’*s assumption that he takes his behavior. Then employee *i* measures the perceived benefit gap between the two situations: when the perceived gap that employee *i* can generate by acting according to his own behavioral strategy is lower than the perceived gap that employee *i* assumes he can generate by taking his own action based on employee *j*’s position, he is influenced by employee *j* and acts in accordance with employee *j*’s approach in the next game; otherwise he determines to take his own behavioral orientation position. In addition, the value used in physics to correspond to the inverse temperature also provides a measure of the strength of natural selection in this study, which can indicate the influence of external bad factors on employees’ behavioral choices. If this is applied, then it means that employees are less sensitive to the difference in perceived benefits, and the behavioral strategies that can obtain higher perceived benefits do not have obvious advantages. This study will follow previous research and set the value to 0.1, which means that employee *i* is more likely to imitate the action stance and behavioral strategy of employee *j* with better perceived benefits.

#### Deviant innovative behaviors of insider subordinates

The high risk-taking trait *insider subordinate* compares the perceived benefits of performing deviant innovative behaviors with the assumption that he is a low risk-taking trait *insider subordinate* who performs or does not perform deviant innovative behaviors, and assuming that high risk-taking trait *insider subordinates* do not perform deviant innovative behaviors, and assuming that high internal control personality *outsider subordinates* do or do not perform deviant innovative behaviors, and assuming that low internal control personality *outsider subordinates* do or do not perform deviant innovative behaviors. We compare perceived benefits to decide whether you need to make a transition in organizational membership or behavioral adjustment. The specific probability is shown in Equation (24) to Equation (30):


(24) $$\text{p}\left({\text{s}}_{\text{ICH}}\leftarrow {\text{s}}_{\text{ICL}}\right)=\frac{1}{1+\text{exp}\left[-\frac{{\text{f}}_{3}^{\text{ICL}}\left(\text{m}\right)-{\text{f}}_{3}^{\text{ICH}}\left(\text{m}\right)}{\kappa }\right]}$$

(25) $$\text{p}\left({\text{s}}_{\text{ICH}}\leftarrow {\text{s}}_{\text{INH}}\right)=\frac{1}{1+\text{exp}\left[-\frac{{\text{f}}_{3}^{\text{INH}}\left(\text{m}\right)-{\text{f}}_{3}^{\text{ICH}}\left(\text{m}\right)}{\kappa }\right]}$$

(26) $$\text{p}\left({\text{s}}_{\text{ICH}}\leftarrow {\text{s}}_{\text{INL}}\right)=\frac{1}{1+\text{exp}\left[-\frac{{\text{f}}_{3}^{\text{INL}}\left(\text{m}\right)-{\text{f}}_{3}^{\text{ICH}}\left(\text{m}\right)}{\kappa }\right]}$$

(27) $$\text{p}\left({\text{s}}_{\text{I}\mathrm{CH}}\leftarrow {\text{s}}_{\text{OCH}}\right)=\frac{1}{1+\text{exp}\left[-\frac{{\text{f}}_{3}^{\text{OCH}}\left(\text{m}\right)-{\text{f}}_{3}^{\text{ICH}}\left(\text{m}\right)}{\kappa }\right]}$$

(28) $$\text{p}\left({\text{s}}_{\text{ICH}}\leftarrow {\text{s}}_{\text{OCL}}\right)=\frac{1}{1+\text{exp}\left[-\frac{{\text{f}}_{3}^{\text{OCL}}\left(\text{m}\right)-{\text{f}}_{3}^{\text{ICH}}\left(\text{m}\right)}{\kappa }\right]}$$

(29) $$\text{p}\left({\text{s}}_{\text{ICH}}\leftarrow {\text{s}}_{\text{ONH}}\right)=\frac{1}{1+\text{exp}\left[-\frac{{\text{f}}_{3}^{\text{ONH}}\left(\text{m}\right)-{\text{f}}_{3}^{\text{ICH}}\left(\text{m}\right)}{\kappa }\right]}$$

(30) $$\text{p}\left({\text{s}}_{\text{ICH}}\leftarrow {\text{s}}_{\text{ONL}}\right)=\frac{1}{1+\text{exp}\left[-\frac{{\text{f}}_{3}^{\text{ONL}}\left(\text{m}\right)-{\text{f}}_{3}^{\text{ICH}}\left(\text{m}\right)}{\kappa }\right]}$$

The low risk-taking trait *insider subordinate* compares the perceived benefits of performing deviant innovative behaviors with the assumption that he is a high risk-taking trait *insider subordinate* who performs or does not perform deviant innovative behaviors, and assuming that low risk-taking trait *insider subordinates* do not perform deviant innovative behaviors, and assuming that low internal control personality *outsider subordinates* do or do not perform deviant innovative behaviors, and assuming that high internal control personality *outsider subordinates* do or do not perform deviant innovative behaviors. We compare perceived benefits to decide whether one needs to make a transition in organizational membership or behavioral adjustment. The specific probability is revealed as Equation (31) to Equation (37):


(31) $$\text{p}\left({\text{s}}_{\text{ICL}}\leftarrow {\text{s}}_{\text{ICH}}\right)=\frac{1}{1+\text{exp}\left[-\frac{{\text{f}}_{3}^{\text{ICH}}\left(\text{m}\right)-{\text{f}}_{3}^{\text{ICL}}\left(\text{m}\right)}{\kappa }\right]}$$

(32) $$\text{p}\left({\text{s}}_{\text{ICL}}\leftarrow {\text{s}}_{\text{INH}}\right)=\frac{1}{1+\text{exp}\left[-\frac{{\text{f}}_{3}^{\text{INH}}\left(\text{m}\right)-{\text{f}}_{3}^{\text{ICL}}\left(\text{m}\right)}{\kappa }\right]}$$

(33) $$\text{p}\left({\text{s}}_{\text{ICL}}\leftarrow {\text{s}}_{\text{INL}}\right)=\frac{1}{1+\text{exp}\left[-\frac{{\text{f}}_{3}^{\text{INL}}\left(\text{m}\right)-{\text{f}}_{3}^{\text{ICL}}\left(\text{m}\right)}{\kappa }\right]}$$

(34) $$\text{p}\left({\text{s}}_{\text{ICL}}\leftarrow {\text{s}}_{\text{OCH}}\right)=\frac{1}{1+\text{exp}\left[-\frac{{\text{f}}_{3}^{\text{OCH}}\left(\text{m}\right)-{\text{f}}_{3}^{\text{ICL}}\left(\text{m}\right)}{\kappa }\right]}$$

(35) $$\text{p}\left({\text{s}}_{\text{ICL}}\leftarrow {\text{s}}_{\text{OCL}}\right)=\frac{1}{1+\text{exp}\left[-\frac{{\text{f}}_{3}^{\text{OCL}}\left(\text{m}\right)-{\text{f}}_{3}^{\text{ICL}}\left(\text{m}\right)}{\kappa }\right]}$$

(36) $$\text{p}\left({\text{s}}_{\text{ICL}}\leftarrow {\text{s}}_{\text{ONH}}\right)=\frac{1}{1+\text{exp}\left[-\frac{{\text{f}}_{3}^{\text{ONH}}\left(\text{m}\right)-{\text{f}}_{3}^{\text{ICL}}\left(\text{m}\right)}{\kappa }\right]}$$

(37) $$\text{p}\left({\text{s}}_{\text{ICL}}\leftarrow {\text{s}}_{\text{ONL}}\right)=\frac{1}{1+\text{exp}\left[-\frac{{\text{f}}_{3}^{\text{ONL}}\left(\text{m}\right)-{\text{f}}_{3}^{\text{ICL}}\left(\text{m}\right)}{\kappa }\right]}$$

According to the inference of the third stage, the perceived benefits of “low risk-taking trait *insider subordinates* who do not perform deviant innovative behaviors are usually higher than those who perform deviant innovative behaviors”, that is, the viewpoint of ${\text{f}}_{3}^{\text{INL}}\left(\text{m}\right)>{\text{f}}_{3}^{\text{ICL}}\left(\text{m}\right)$, in the above, only Equation (33) indicates that, under the condition of low risk-taking characteristics, *insider subordinates* will change from implementing deviant innovative behaviors to not implement deviant innovation behavior changes.

#### Normal innovative behaviors of insider subordinates

The high risk-taking trait *insider subordinate* takes the perceived benefits from deviant innovation behavior and assumes as a low risk-taking trait *insider subordinate*, assume oneself as a high or low risk trait *insider subordinate* do not perform deviant innovation behavior, assume oneself as a high or low internal control type of personality *outsider subordinate* to implement deviant innovative behavior and assume that oneself as high or low internal control type of personality *outsider subordinate* do not implement deviant innovation behavior. We compare perceived benefits to decide whether there needs to be a transition in organizational membership or behavioral adjustment. The specific probability is revealed as Equation (38) to Equation (44):


(38) $$\text{p}\left({\text{s}}_{\text{INH}}\leftarrow {\text{s}}_{\text{ICH}}\right)=\frac{1}{1+\text{exp}\left[-\frac{{\text{f}}_{3}^{\text{ICH}}\left(\text{m}\right)-{\text{f}}_{3}^{\text{INH}}\left(\text{m}\right)}{\kappa }\right]}$$

(39) $$\text{p}\left({\text{s}}_{\text{INH}}\leftarrow {\text{s}}_{\text{ICL}}\right)=\frac{1}{1+\text{exp}\left[-\frac{{\text{f}}_{3}^{\text{ICL}}\left(\text{m}\right)-{\text{f}}_{3}^{\text{INH}}\left(\text{m}\right)}{\kappa }\right]}$$

(40) $$\text{p}\left({\text{s}}_{\text{INH}}\leftarrow {\text{s}}_{\text{INL}}\right)=\frac{1}{1+\text{exp}\left[-\frac{{\text{f}}_{3}^{\text{INL}}\left(\text{m}\right)-{\text{f}}_{3}^{\text{INH}}\left(\text{m}\right)}{\kappa }\right]}$$

(41) $$\text{p}\left({\text{s}}_{\text{INH}}\leftarrow {\text{s}}_{\text{OCH}}\right)=\frac{1}{1+\text{exp}\left[-\frac{{\text{f}}_{3}^{\text{OCH}}\left(\text{m}\right)-{\text{f}}_{3}^{\text{INH}}\left(\text{m}\right)}{\kappa }\right]}$$

(42) $$\text{p}\left({\text{s}}_{\text{INH}}\leftarrow {\text{s}}_{\text{OCL}}\right)=\frac{1}{1+\text{exp}\left[-\frac{{\text{f}}_{3}^{\text{OCL}}\left(\text{m}\right)-{\text{f}}_{3}^{\text{INH}}\left(\text{m}\right)}{\kappa }\right]}$$

(43) $$\text{p}\left({\text{s}}_{\text{INH}}\leftarrow {\text{s}}_{\text{ONH}}\right)=\frac{1}{1+\text{exp}\left[-\frac{{\text{f}}_{3}^{\text{ONH}}\left(\text{m}\right)-{\text{f}}_{3}^{\text{INH}}\left(\text{m}\right)}{\kappa }\right]}$$

(44) $$\text{p}\left({\text{s}}_{\text{INH}}\leftarrow {\text{s}}_{\text{ONL}}\right)=\frac{1}{1+\text{exp}\left[-\frac{{\text{f}}_{3}^{\text{ONL}}\left(\text{m}\right)-{\text{f}}_{3}^{\text{INH}}\left(\text{m}\right)}{\kappa }\right]}$$

The low risk-taking trait *insider subordinate* compares the perceived benefits of not engaging in deviant innovation behaviors with the assumption that he is a high risk-taking trait *insider subordinate* who performs or does not perform deviant innovative behaviors, and assuming that low risk-taking trait *insider subordinates* to perform deviant innovative behaviors, and assuming that low internal control personality *outsider subordinates* perform or do not perform deviant innovative behaviors, and assuming that high internal control personality *outsider subordinates* perform or do not perform deviant innovative behaviors. We compare perceived benefits to decide whether there needs to be a transition in organizational membership or behavioral adjustment. The specific probability is revealed as Equation (45) to Equation (51):


(45) $$\text{p}\left({\text{s}}_{\text{INL}}\leftarrow {\text{s}}_{\text{ICH}}\right)=\frac{1}{1+\text{exp}\left[-\frac{{\text{f}}_{3}^{\text{ICH}}\left(\text{m}\right)-{\text{f}}_{3}^{\text{INL}}\left(\text{m}\right)}{\kappa }\right]}$$

(46) $$\text{p}\left({\text{s}}_{\text{INL}}\leftarrow {\text{s}}_{\text{ICL}}\right)=\frac{1}{1+\text{exp}\left[-\frac{{\text{f}}_{3}^{\text{ICL}}\left(\text{m}\right)-{\text{f}}_{3}^{\text{INL}}\left(\text{m}\right)}{\kappa }\right]}$$

(47) $$\text{p}\left({\text{s}}_{\text{INL}}\leftarrow {\text{s}}_{\text{INH}}\right)=\frac{1}{1+\text{exp}\left[-\frac{{\text{f}}_{3}^{\text{INH}}\left(\text{m}\right)-{\text{f}}_{3}^{\text{INL}}\left(\text{m}\right)}{\kappa }\right]}$$

(48) $$\text{p}\left({\text{s}}_{\text{INL}}\leftarrow {\text{s}}_{\text{OCH}}\right)=\frac{1}{1+\text{exp}\left[-\frac{{\text{f}}_{3}^{\text{OCH}}\left(\text{m}\right)-{\text{f}}_{3}^{\text{INL}}\left(\text{m}\right)}{\kappa }\right]}$$

(49) $$\text{p}\left({\text{s}}_{\text{INL}}\leftarrow {\text{s}}_{\text{OCL}}\right)=\frac{1}{1+\text{exp}\left[-\frac{{\text{f}}_{3}^{\text{OCL}}\left(\text{m}\right)-{\text{f}}_{3}^{\text{INL}}\left(\text{m}\right)}{\kappa }\right]}$$

(50) $$\text{p}\left({\text{s}}_{\text{INL}}\leftarrow {\text{s}}_{\text{ONH}}\right)=\frac{1}{1+\text{exp}\left[-\frac{{\text{f}}_{3}^{\text{ONH}}\left(\text{m}\right)-{\text{f}}_{3}^{\text{INL}}\left(\text{m}\right)}{\kappa }\right]}$$

(51) $$\text{p}\left({\text{s}}_{\text{INL}}\leftarrow {\text{s}}_{\text{ONL}}\right)=\frac{1}{1+\text{exp}\left[-\frac{{\text{f}}_{3}^{\text{ONL}}\left(\text{m}\right)-{\text{f}}_{3}^{\text{INL}}\left(\text{m}\right)}{\kappa }\right]}$$

According to the third stage, the perceived benefit from the high-risk trait *insider subordinates* is usually higher than those who without performing the deviate innovation behavior and it is equal to ${\text{f}}_{3}^{\text{ICH}}\left(\text{m}\right)>{\text{f}}_{3}^{\text{INH}}\left(\text{m}\right)$. The above is only established in Equation (38), which shows that *insider subordinates* will change from not implementing deviant innovative behaviors to implementing deviant innovative behaviors.

#### Deviant innovative behaviors of outsider subordinates

The high internal control personality *outsider subordinates* compare the perceived benefits of deviant innovative behaviors with the assumption that they are high risk-taking *insider subordinates* to perform or not to perform deviant innovative behaviors, and assuming that low risk-taking *insider subordinates* to perform or not to perform deviant innovative behaviors, and assuming that low internal control personality *outsider subordinates* to perform or not to perform deviant innovative behaviors, and assuming that high internal control personality *outsider subordinates* do not perform deviant innovative behaviors. We compare perceived benefits to decide whether there needs to be a transition in organizational membership or behavioral adjustment. The specific probability is revealed as Equation (52) to Equation (58):


(52) $$\text{p}\left({\text{s}}_{\text{OCH}}\leftarrow {\text{s}}_{\text{ICH}}\right)=\frac{1}{1+\text{exp}\left[-\frac{{\text{f}}_{3}^{\text{ICH}}\left(\text{m}\right)-{\text{f}}_{3}^{\text{OCH}}\left(\text{m}\right)}{\kappa }\right]}$$

(53) $$\text{p}\left({\text{s}}_{\text{OCH}}\leftarrow {\text{s}}_{\text{ICL}}\right)=\frac{1}{1+\text{exp}\left[-\frac{{\text{f}}_{3}^{\text{ICL}}\left(\text{m}\right)-{\text{f}}_{3}^{\text{OCH}}\left(\text{m}\right)}{\kappa }\right]}$$

(54) $$\text{p}\left({\text{s}}_{\text{OCH}}\leftarrow {\text{s}}_{\text{INH}}\right)=\frac{1}{1+\text{exp}\left[-\frac{{\text{f}}_{3}^{\text{INH}}\left(\text{m}\right)-{\text{f}}_{3}^{\text{OCH}}\left(\text{m}\right)}{\kappa }\right]}$$

(55) $$\text{p}\left({\text{s}}_{\text{OCH}}\leftarrow {\text{s}}_{\text{INL}}\right)=\frac{1}{1+\text{exp}\left[-\frac{{\text{f}}_{3}^{\text{INL}}\left(\text{m}\right)-{\text{f}}_{3}^{\text{OCH}}\left(\text{m}\right)}{\kappa }\right]}$$

(56) $$\text{p}\left({\text{s}}_{\text{OCH}}\leftarrow {\text{s}}_{\text{OCL}}\right)=\frac{1}{1+\text{exp}\left[-\frac{{\text{f}}_{3}^{\text{OCL}}\left(\text{m}\right)-{\text{f}}_{3}^{\text{OCH}}\left(\text{m}\right)}{\kappa }\right]}$$

(57) $$\text{p}\left({\text{s}}_{\text{OCH}}\leftarrow {\text{s}}_{\text{ONH}}\right)=\frac{1}{1+\text{exp}\left[-\frac{{\text{f}}_{3}^{\text{ONH}}\left(\text{m}\right)-{\text{f}}_{3}^{\text{OCH}}\left(\text{m}\right)}{\kappa }\right]}$$

(58) $$\text{p}\left({\text{s}}_{\text{OCH}}\leftarrow {\text{s}}_{\text{ONL}}\right)=\frac{1}{1+\text{exp}\left[-\frac{{\text{f}}_{3}^{\text{ONL}}\left(\text{m}\right)-{\text{f}}_{3}^{\text{OCH}}\left(\text{m}\right)}{\kappa }\right]}$$

The low internal control personality *outsider subordinates* compare the perceived benefits of deviant innovative behaviors with the assumption that they are high risk-taking *insider subordinates* to perform or not to perform deviant innovative behaviors, and assuming that low risk-taking *insider subordinates* do or do not perform deviant innovative behaviors, and assuming that high internal control personality *outsider subordinates* do or do not perform deviant innovative behaviors, and assuming that low internal control personality *outsider subordinates* do not perform deviant innovative behaviors. We compare perceived benefits to decide whether there needs to be a transition in organizational membership or behavioral adjustment. The specific probability is revealed as Equation (59) to Equation (65):


(59) $$\text{p}\left({\text{s}}_{\text{OCL}}\leftarrow {\text{s}}_{\text{ICH}}\right)=\frac{1}{1+\text{exp}\left[-\frac{{\text{f}}_{3}^{\text{ICH}}\left(\text{m}\right)-{\text{f}}_{3}^{\text{OCL}}\left(\text{m}\right)}{\kappa }\right]}$$

(60) $$\text{p}\left({\text{s}}_{\text{OCL}}\leftarrow {\text{s}}_{\text{ICL}}\right)=\frac{1}{1+\text{exp}\left[-\frac{{\text{f}}_{3}^{\text{ICL}}\left(\text{m}\right)-{\text{f}}_{3}^{\text{OCL}}\left(\text{m}\right)}{\kappa }\right]}$$

(61) $$\text{p}\left({\text{s}}_{\text{OCL}}\leftarrow {\text{s}}_{\text{INH}}\right)=\frac{1}{1+\text{exp}\left[-\frac{{\text{f}}_{3}^{\text{INH}}\left(\text{m}\right)-{\text{f}}_{3}^{\text{OCL}}\left(\text{m}\right)}{\kappa }\right]}$$

(62) $$\text{p}\left({\text{s}}_{\text{OCL}}\leftarrow {\text{s}}_{\text{INL}}\right)=\frac{1}{1+\text{exp}\left[-\frac{{\text{f}}_{3}^{\text{INL}}\left(\text{m}\right)-{\text{f}}_{3}^{\text{OCL}}\left(\text{m}\right)}{\kappa }\right]}$$

(63) $$\text{p}\left({\text{s}}_{\text{OCL}}\leftarrow {\text{s}}_{\text{OCH}}\right)=\frac{1}{1+\text{exp}\left[-\frac{{\text{f}}_{3}^{\text{OCH}}\left(\text{m}\right)-{\text{f}}_{3}^{\text{OCL}}\left(\text{m}\right)}{\kappa }\right]}$$

(64) $$\text{p}\left({\text{s}}_{\text{OCL}}\leftarrow {\text{s}}_{\text{ONH}}\right)=\frac{1}{1+\text{exp}\left[-\frac{{\text{f}}_{3}^{\text{ONH}}\left(\text{m}\right)-{\text{f}}_{3}^{\text{OCL}}\left(\text{m}\right)}{\kappa }\right]}$$

(65) $$\text{p}\left({\text{s}}_{\text{OCL}}\leftarrow {\text{s}}_{\text{ONL}}\right)=\frac{1}{1+\text{exp}\left[-\frac{{\text{f}}_{3}^{\text{ONL}}\left(\text{m}\right)-{\text{f}}_{3}^{\text{OCL}}\left(\text{m}\right)}{\kappa }\right]}$$

According to the third stage, the perceived income generated by the low internal control personality *outsider subordinate* not performing the deviant innovation behavior is usually higher than those who are performing the deviant innovation behavior. This is equal to ${\text{f}}_{3}^{\text{ONL}}\left(\text{m}\right)>{\text{f}}_{3}^{\text{OCL}}\left(\text{m}\right)$. In the above, only Equation (65) is established, indicating that *outsider subordinate* will change from implementing deviant innovative behaviors to not implementing deviant innovative behaviors under the condition of low internal control personality.

#### Normal innovative behaviors of outsider subordinates

The high internal control personality *outsider subordinates* compare the perceived benefits of not implementing deviant innovative behaviors with the assumption that they are high risk-taking *insider subordinates* to perform or not to perform deviant innovative behaviors, and assuming that low risk-taking *insider subordinates* perform or do not perform deviant innovative behaviors, and assuming that low internal control personality *outsider subordinates* perform or do not perform deviant innovative behaviors, and assuming that high internal control personality *outsider subordinates* do not perform deviant innovative behaviors. We compare perceived benefits to decide whether there needs to be a transition in organizational membership or behavioral adjustment. The specific probability is revealed in Equation (66) to Equation (72):


(66) $$\text{p}\left({\text{s}}_{\text{ONH}}\leftarrow {\text{s}}_{\text{ICH}}\right)=\frac{1}{1+\text{exp}\left[-\frac{{\text{f}}_{3}^{\text{ICH}}\left(\text{m}\right)-{\text{f}}_{3}^{\text{ONH}}\left(\text{m}\right)}{\kappa }\right]}$$

(67) $$\text{p}\left({\text{s}}_{\text{ONH}}\leftarrow {\text{s}}_{\text{ICL}}\right)=\frac{1}{1+\text{exp}\left[-\frac{{\text{f}}_{3}^{\text{ICL}}\left(\text{m}\right)-{\text{f}}_{3}^{\text{ONH}}\left(\text{m}\right)}{\kappa }\right]}$$

(68) $$\text{p}\left({\text{s}}_{\text{ONH}}\leftarrow {\text{s}}_{\text{INH}}\right)=\frac{1}{1+\text{exp}\left[-\frac{{\text{f}}_{3}^{\text{INH}}\left(\text{m}\right)-{\text{f}}_{3}^{\text{ONH}}\left(\text{m}\right)}{\kappa }\right]}$$

(69) $$\text{p}\left({\text{s}}_{\text{ONH}}\leftarrow {\text{s}}_{\text{INL}}\right)=\frac{1}{1+\text{exp}\left[-\frac{{\text{f}}_{3}^{\text{INL}}\left(\text{m}\right)-{\text{f}}_{3}^{\text{ONH}}\left(\text{m}\right)}{\kappa }\right]}$$

(70) $$\text{p}\left({\text{s}}_{\text{ONH}}\leftarrow {\text{s}}_{\text{OCH}}\right)=\frac{1}{1+\text{exp}\left[-\frac{{\text{f}}_{3}^{\text{OCH}}\left(\text{m}\right)-{\text{f}}_{3}^{\text{ONH}}\left(\text{m}\right)}{\kappa }\right]}$$

(71) $$\text{p}\left({\text{s}}_{\text{ONH}}\leftarrow {\text{s}}_{\text{OCL}}\right)=\frac{1}{1+\text{exp}\left[-\frac{{\text{f}}_{3}^{\text{OCL}}\left(\text{m}\right)-{\text{f}}_{3}^{\text{ONH}}\left(\text{m}\right)}{\kappa }\right]}$$

(72) $$\text{p}\left({\text{s}}_{\text{ONH}}\leftarrow {\text{s}}_{\text{ONL}}\right)=\frac{1}{1+\text{exp}\left[-\frac{{\text{f}}_{3}^{\text{ONL}}\left(\text{m}\right)-{\text{f}}_{3}^{\text{ONH}}\left(\text{m}\right)}{\kappa }\right]}$$

The low internal control personality *outsider subordinates* compare the perceived benefits of not implementing deviant innovative behaviors with the assumption that they are high risk-taking *insider subordinates* to perform or not to perform deviant innovative behaviors, and assuming that low risk-taking *insider subordinates* do or do not perform deviant innovative behaviors, and assuming that high internal control personality *outsider subordinates* do or do not perform deviant innovative behaviors, and assuming that low internal control personality *outsider subordinates* perform deviant innovative behaviors. We compare perceived benefits to decide whether there needs to be a transition in organizational membership or behavioral adjustment. The specific probability is revealed as Equation (73) to Equation (79):


(73) $$\text{p}\left({\text{s}}_{\text{ONL}}\leftarrow {\text{s}}_{\text{ICH}}\right)=\frac{1}{1+\text{exp}\left[-\frac{{\text{f}}_{3}^{\text{ICH}}\left(\text{m}\right)-{\text{f}}_{3}^{\text{ONL}}\left(\text{m}\right)}{\kappa }\right]}$$

(74) $$\text{p}\left({\text{s}}_{\text{ONL}}\leftarrow {\text{s}}_{\text{ICL}}\right)=\frac{1}{1+\text{exp}\left[-\frac{{\text{f}}_{3}^{\text{ICL}}\left(\text{m}\right)-{\text{f}}_{3}^{\text{ONL}}\left(\text{m}\right)}{\kappa }\right]}$$

(75) $$\text{p}\left({\text{s}}_{\text{ONL}}\leftarrow {\text{s}}_{\text{INH}}\right)=\frac{1}{1+\text{exp}\left[-\frac{{\text{f}}_{3}^{\text{INH}}\left(\text{m}\right)-{\text{f}}_{3}^{\text{ONL}}\left(\text{m}\right)}{\kappa }\right]}$$

(76) $$\text{p}\left({\text{s}}_{\text{ONL}}\leftarrow {\text{s}}_{\text{INL}}\right)=\frac{1}{1+\text{exp}\left[-\frac{{\text{f}}_{3}^{\text{INL}}\left(\text{m}\right)-{\text{f}}_{3}^{\text{ONL}}\left(\text{m}\right)}{\kappa }\right]}$$

(77) $$\text{p}\left({\text{s}}_{\text{ONL}}\leftarrow {\text{s}}_{\text{OCH}}\right)=\frac{1}{1+\text{exp}\left[-\frac{{\text{f}}_{3}^{\text{OCH}}\left(\text{m}\right)-{\text{f}}_{3}^{\text{ONL}}\left(\text{m}\right)}{\kappa }\right]}$$

(78) $$\text{p}\left({\text{s}}_{\text{ONL}}\leftarrow {\text{s}}_{\text{OCL}}\right)=\frac{1}{1+\text{exp}\left[-\frac{{\text{f}}_{3}^{\text{OCL}}\left(\text{m}\right)-{\text{f}}_{3}^{\text{ONL}}\left(\text{m}\right)}{\kappa }\right]}$$

(79) $$\text{p}\left({\text{s}}_{\text{ONL}}\leftarrow {\text{s}}_{\text{ONH}}\right)=\frac{1}{1+\text{exp}\left[-\frac{{\text{f}}_{3}^{\text{ONH}}\left(\text{m}\right)-{\text{f}}_{3}^{\text{ONL}}\left(\text{m}\right)}{\kappa }\right]}$$

According to the third stage, the perceived income generated by the implementation of deviant innovation behavior with high internal control personality *outsider subordinate* is usually higher than those who are not performing deviant innovation behavior. This is equal to ${\text{f}}_{3}^{\text{OCH}}\left(\text{m}\right)>{\text{f}}_{3}^{\mathrm{ON}\text{H}}\left(\text{m}\right)$. In the above, only Equation (70) is established, indicating that *outsider subordinates* will change from never implementing deviant innovation behavior to implementing deviant innovation behavior.

### Simulation analyses

The differential treatment of employees by differential order leaders will divide the different distribution types of *insider subordinates* and *outsider subordinates* in the organizational relationship, and this provides information clues about the difference between their role allocation and identity. The different combination forms of i*nsider subordinate* and *outsider subordinate* will then affect their cognitive attitude and emotional relationship, which is an important situational factor for interpreting employees’ behavior response (Jiang and Zheng, 2014). In this study, the number of employees is set as *N* in the initial organizational network structure; the proportion of *insider subordinates* is ρ_*I*_, the proportion of *outsider subordinates* is ρ_*O*_ and ρ_I_ + ρ_O_ = 1. The degree distribution of the organizational network structure is P(q) = 2g^2^q^−α^, the average degree *q* = 6, the minimum degree *g* = 3, the power exponent = 2.1. The Monte Carlo method is used to perform a game model simulation process that differential order leadership affects the de-innovation behavior of employees, and that the average value of 200 independent runs is used as the final result to avoid random influence effects.

### Differential leadership and deviant innovative behaviors of insider subordinates

**FIGURE 1:**
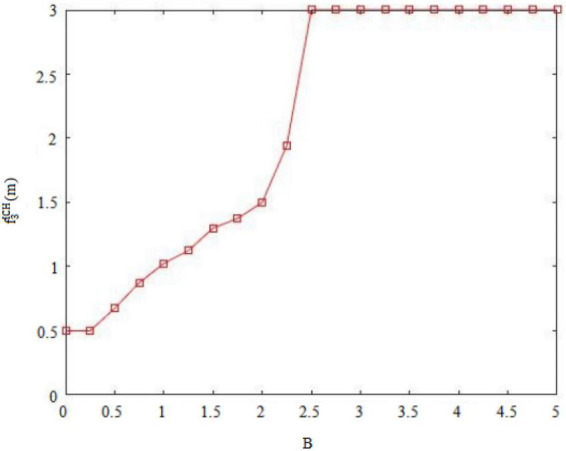
Deviant innovative behaviors and perceived benefits of high risk-taking traits of “insider subordinates.”

**FIGURE 2:**
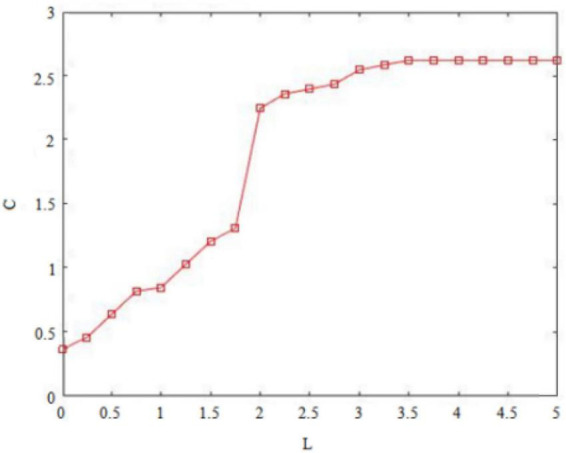
Differential leadership and the psychological capital of “insider subordinates.”

How the psychological capital from the perspective of *insider subordinates* changes with the biased treatment of different order leaders, how the perceived benefits of *insider subordinates* change with the risky traits, and how the probability of implementing deviant innovation behavior changes with the supplementary expectation of perceived return to psychological capital will be explained in this study. Firstly, it is known that *insider subordinates* find it easy to form a unique cognitive and emotional meaning within the organization after understanding the biased treatment of differential order leaders, and this is equal to additional psychological capital focus that occurs due to the *insider subordinates* role level given by the differential order leadership (Fu et al., 2021). Figure 1 shows a simulation diagram of how the *insider subordinates’* psychological capital C changes with the differential leadership level L. It is known that differential leaders will give *insider subordinates* trust in terms of communication, promotion, rewards, tolerance. Meanwhile, *insider subordinates* have an increasing of psychological capital. As shown in Figure 2, *insider subordinate* psychological capital C and differential order leadership level L are positively correlated. The higher the level of differential leadership, the more psychological capital the *insider subordinates* can gain.

**FIGURE 3:**
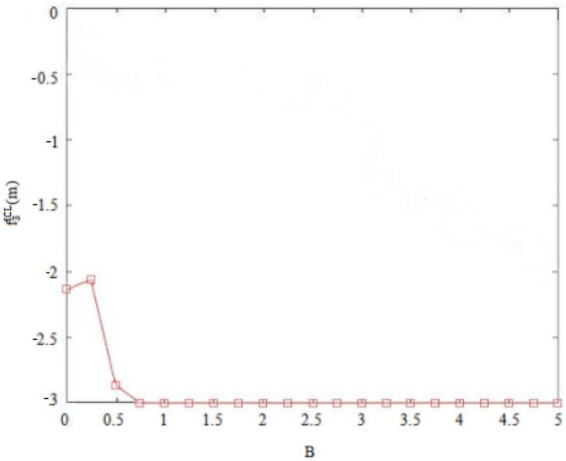
Deviant innovative behaviors and perceived benefits of low risk-taking traits of “insider subordinates.”

Secondly, risk-taking traits can cause fluctuations in the original balance of pros and cons and general behavioral orientations of *insider subordinates* (Venkatraman, 1991). For example, high risk-taking traits of *insider subordinates* focus on the appreciation of rights and interests, so that they will abandon the dangerous concerns hidden behind their behaviors, and may pass on perceived benefits through the implementation of deviant innovative behaviors. Conversely, the low risk-taking traits of *insider subordinates* prefer to pursue stability and pay attention to maintaining vested interests, deepen their cognition of the “deviant” nature of deviant innovative behaviors, and may convey doubts about perceived benefits through the implementation of deviant innovative behaviors. Figures 1, 3 therefore plot the situations in which the two types of employees with *insider subordinates’* high risk-taking traits and low risk-taking traits implement deviant innovative behavior B and can capture perceived benefits $f_{3}^{\text{ICH}}\left(\text{m}\right)\text{ or }{\text{f}}_{3}^{\text{ICL}}\left(\text{m}\right)$.

**FIGURE 4:**
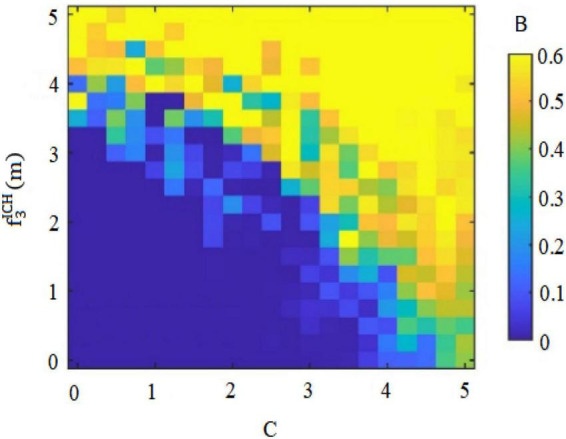
Psychological capital, perceived benefits and deviant innovation behavior (high risk-taking traits).

Of these, Figure 3 shows the perceived benefits that can be obtained by high-risk-taking trait *subordinates inside the circle* when performing deviant innovative behaviors. Figure 4 shows the perceived benefits that can be obtained by low-risk-taking trait *subordinates inside the circle* who carry out deviant innovative behaviors. Through the paired observations in Figures 3, 4, it can be observed that the perceived benefits of employees’ deviant innovative behaviors show a completely different trend according to the differing levels of risk-taking traits.

In Figure 3, the high risk-taking traits of *insider subordinates* are directly proportional to the perceived benefits that they can obtain when they perform deviant innovative behaviors. In Figure 4, the low risk-taking traits of *insider subordinates* are inversely proportional to their perceived benefits. The results show that the perceived benefits of high-risk *insider subordinates* derived from their deviant innovation behavior are significantly higher than those of low-risk *insider subordinates* from their deviant innovation behavior.

**FIGURE 5:**
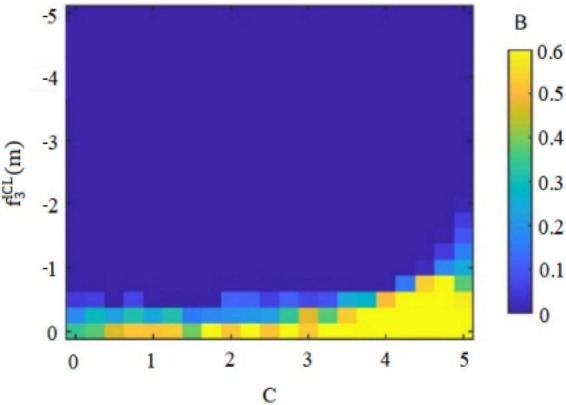
Psychological capital, perceived benefit, and deviant innovative behavior (low risk-taking trait).

Thirdly, whether *subordinates inside the circle* can form perceived benefits for deviant innovative behavior, and whether the perceived benefits can effectively supplement the psychological capital formed by the biased treatment of differential leadership, are the initiating factors of whether *subordinates inside the circle* will carry out deviant innovative behavior. This study therefore analyzed the possibility B of *subordinates inside the circle* performing deviant innovative behavior under the dual effect of the negative treatment of differential leadership to inhibit the psychological capital C of *insider subordinates* and the perceived benefits of deviant innovative behavior $f_{3}^{\text{ICH}}\left(\text{m}\right)$ or $f_{3}^{\text{ICL}}\left(\text{m}\right).$ The dual display is shown in Figures 4, 5.

In Figure 4, under the condition of high risk-taking characteristics, the possibility of *subordinates inside the circle* performing deviant innovative behaviors increases with the addition of *subordinates’ inside the circle* psychological capital, and also with the implementation of deviant innovative behaviors. If the perceived benefit increases, the possibility of executing deviant innovative behaviors is affected by the interaction between psychological capital and perceived benefit.

In Figure 5, under the condition of low risk-taking traits, the possibility of *subordinates inside the circle* performing deviant innovative behaviors increases with the addition of *subordinates’ inside the circle* psychological capital. However, the perceived benefits of *subordinates inside the circle* performing deviant innovative behaviors show a decreasing trend, and the perceived benefits of deviant innovative behaviors are the opposite. This will weaken their psychological capital and curb the possibility of its execution of deviant innovative behaviors.

### Differential leadership and the deviant innovative behavior of “subordinates outside the circle”

This study simulates and interprets how psychological capital changes with the preferential treatment of differential leaders from the perspective of *subordinates outside the circle*, how the perceived benefits of *subordinates outside the circle* performing deviant innovative behaviors change with the internal control personality, and how the probability of *subordinates outside the circle* performing deviant innovative behaviors changes with the supplementary expectations of perceived benefits for psychological capital.

**FIGURE 6:**
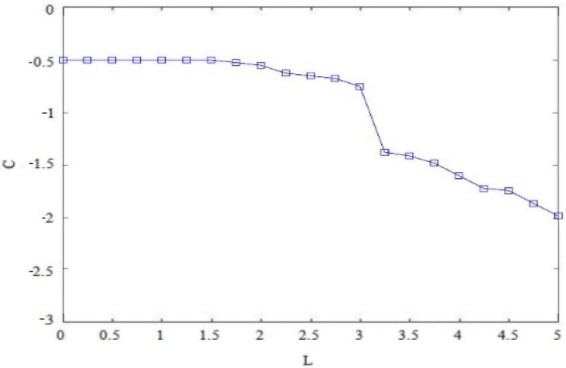
Differential leadership and the psychological capital of “subordinates outside the circle.”

First, it is known that *subordinates outside the circle* are prone to form an inexhaustible cognitive and emotional connotation within the organization after learning about the disadvantaged treatment of differential leadership. Due to the role level of *subordinates outside the circle* that is endowed by differential leadership, it is inevitable that a loss of psychological capital occurs (Guan and Yan, 2021). Figure 6 draws a simulation diagram of how psychological capital C of the *subordinates outside the circle* changes with the differential leadership level L. It is known that differential leaders will give *subordinates outside the circle* preferential treatment in terms of taking care of communication, promotion and rewards, tolerance and trust (Zheng, 2004), which generates a feeling of shock and produces a dissolving effect of psychological capital. Therefore, there is a negative correlation between the psychological capital C of *subordinates outside the circle* and the differential leadership level L. As shown in Figure 6, the higher the differential leadership level, the more collapsed will be the psychological capital of *subordinates outside the circle*.

**FIGURE 7:**
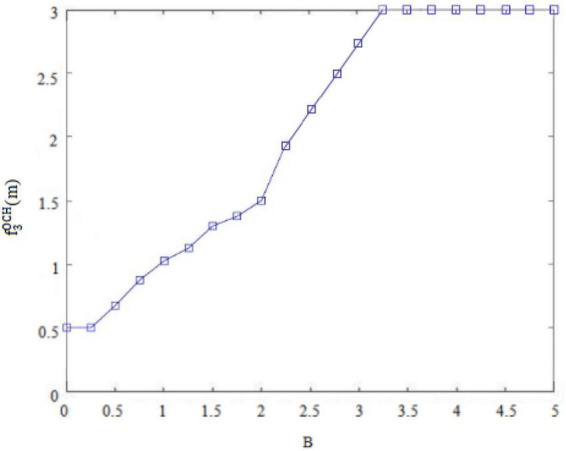
Deviant innovative behaviors and perceived benefits of “outsiders’ subordinates” with high internal control personality.

**FIGURE 8:**
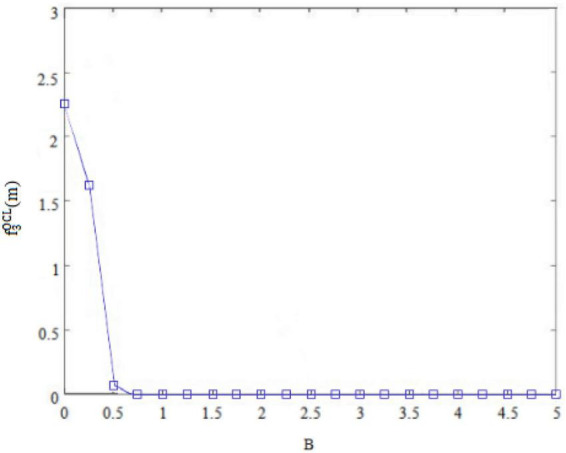
Deviant innovative behaviors and perceived benefits of “outsiders’ subordinates” with low internal control personality.

Second, the internal control personality can interfere with the original balance of success and failure and general behavioral orientation of *subordinates outside the circle* (Joe, 1971). For example, *subordinates outside the circle* with high internal control personality are convinced of their own behavioral abilities, so that they may deliver perceived benefits through deviant innovative behaviors. Conversely, *subordinates outside the circle* with low internal control personality think that they are unable to change rigid reality, and so they will not deliver perceived benefits through deviant innovative behaviors. Figures 7, 8 thus plot the situation where the deviant innovative behavior B of employees with high internal control personality and low internal control personality *subordinates outside the circle* can seize the perceived benefits $f_{3}^{\text{OCH}}\left(\text{m}\right)$ or $f_{3}^{\text{OCL}}\left(\text{m}\right)$. Of these, Figure 7 shows the perceived benefits obtained by *subordinates outside the circle* with high internal control personality from performing deviant innovative behaviors, and Figure 8 shows the perceived benefits obtained by *subordinates outside the circle* with low internal control personality from performing deviant innovative behaviors. Through the paired observations of Figures 7, 8, it can be seen that the perceived benefits of employees’ deviant innovative behaviors show a very different trend with different levels of internal control personality. In the case of Figure 7, the deviant innovative behavior of *subordinates outside the circle* with high internal control personality is directly proportional to the perceived benefits that they can obtain. In the case of Figure 8, the perceived benefit of deviant innovative behavior by *subordinates outside the circle* with low internal control personality is reduced to 0. These results indicate that the perceived benefits of deviant innovative behavior of *subordinates outside the circle* with high internal control personality are significantly higher than those of *subordinates outside the circle* with low internal control personality.

**FIGURE 9:**
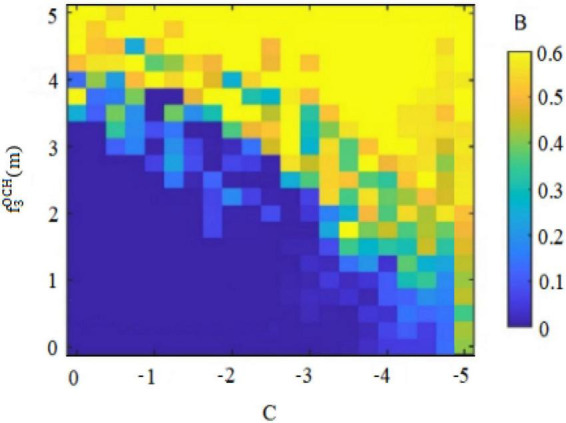
Psychological capital, perceived benefits and deviant innovative behavior (high internal control personality).

**FIGURE 10:**
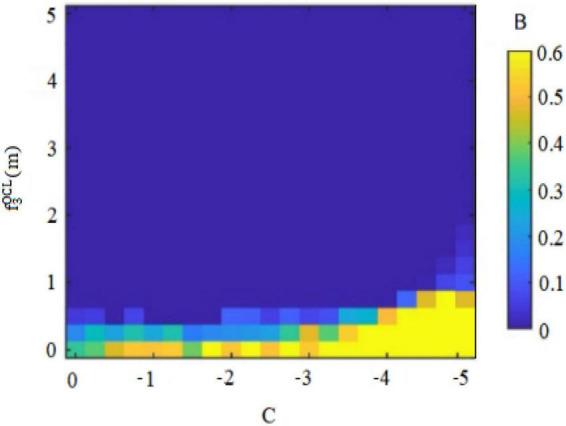
Psychological capital, perceived benefits and deviant innovative behavior (low internal control personality).

Third, whether the *subordinates outside the circle* can form a perceived benefit for deviant innovative behaviors. and whether the perceived benefit can effectively supplement the weak psychological capital caused by the deviant leadership’s negative treatment, are the initiating factors of whether *subordinates outside the circle* will carry out deviant innovative behavior. Therefore this study analyzed the possibility B of *outsider subordinates* performing deviant innovative behavior under the dual effect of the negative treatment of differential leadership so as to inhibit the psychological capital C of *outsider subordinates* and the perceived benefits of deviant innovative behavior $f_{3}^{\text{OCH}}\left(\text{m}\right)$ or$f_{3}^{\text{OCL}}\left(\text{m}\right)$. The dual display is shown in Figures 9, 10.

In Figure 9, under the condition of high internal control personality, the possibility of *outsider subordinates* performing deviant innovative behaviors increases with the decrease of their psychological capital. It also increases with the increase of the perceived benefits of the deviant innovative behavior, so that the possibility of the deviant innovative behavior is affected by the interaction between psychological capital and perceived benefits.

In Figure 10, under the condition of low internal control personality, the possibility of *outsider subordinates* executing deviant innovative behaviors increases with the decline of their psychological capital. However, the perceived benefits of the deviant innovative behavior of *subordinates outside the circle* show a gentle trend, so the perceived benefits of their deviant innovative behaviors cannot promote the accumulation of their psychological capital. Therefore, this will restrain the possibility of their deviant innovative behavior.

## Conclusion

This study uses the evolutionary game model to simulate the psychology and behavior course of employees’ deviant innovative behavior in the context of differentiated leadership. On the one hand, whether or not *subordinates inside the circle* with high or low risk characteristics can obtain the perceived benefits from the deviant innovative behavior directly shows that the *subordinates inside the circle* intend to return the increase of psychological capital by the deviant innovative behavior. On the other hand, this study has examined whether *subordinates outside the circle* with high or low level of internal control personality could obtain perceived benefits from their deviant innovative behavior, which directly indicates that *subordinates outside the circle* intended to reverse the psychological capital impairment caused by the deviant leadership’s negative treatment by way of their own deviant innovative behavior. In this section of the study, we take the outsider subordinates with high internal control personality and low internal control personality as the research object, and explore and analyze the behaviors caused by differential leaders’ biased behavior and the reasons for the impairment of psychological capital brought about by deviant innovative behaviors.

### The psychological capital of insider subordinates and outsider subordinates

The game study on the influence of differential leadership on employees’ deviant innovative behavior is a two-dimensional process involving *subordinates inside the circle* and *subordinates outside the circle*, and this needs to be divided into two paths for relatively independent detail development. Leaders divide employees into *subordinates inside the circle* and *subordinates outside the circle* and treat them differently, so that employees will form different internal psychological states (Jiang and Zheng, 2014). As shown in Figures 2, 6, the biased treatment of *subordinates inside the circle* in the face of differential leadership can increase their psychological capital, while the negative treatment of *subordinates outside the circle* in the face of differential leadership will wipe out their psychological capital. It can be seen, then, that the two sides present completely opposite inner states. Therefore, although the deviant innovative behaviors of *subordinates inside the circle* and *subordinates outside the circle* can be stimulated by differential leadership, they have completely different psychological processes due to the different roles and positions in the organization. *Subordinates inside the circle* will not hurt or betray the collaborative relationship with leaders, and are even more likely to go beyond the scope of responsibilities and rules to improve innovation performance, so as to form an excessive response to the biased treatment of differential leaders (Fu et al., 2021). Although *subordinates outside the circle* are not willing to be restrained by the role of differential leaders, they will not easily give up the opportunity to reverse their lost situation, which may cause *subordinates outside the circle* to take deviating behavioral means to deal with the resistance to advance to a certain extent (Guan and Yan, 2021).

### Influence of personality traits on perceived benefits of deviant innovative behaviors

Based on the above findings and arguments, the game reasoning and simulation diagrams of the perceived benefits of deviant innovative behaviors for the high and low risk-taking traits of *subordinates inside the circle* and the high and low internal control personality *subordinates outside the circle* are sufficient to show that the personality traits of employees will affect the perception of innovation of deviant behavior in the current situation (McCrae and Costa, 1997). The risk-taking trait level will affect the perceived benefits generated by the deviant innovative behavior of *insider subordinates* (Venkatraman, 1991). Figure 1 shows that the perceived benefits obtained by the deviant innovative behavior of *insider subordinates* with high risk-taking trait show an upward trend and the value is positive. Figure 3 shows that the perceived benefits of low-risk *insider subordinates* from deviant innovative behavior show a downward trend and a negative value, indicating that high-risk *insider subordinates* are more concerned about the possibility of profit from deviant innovative behavior, while low-risk *insider subordinates* are more afraid of being punished for deviant innovative behaviors. The level of internal control personality will influence the perceived benefits generated by the deviant innovative behavior of *subordinates outside the circle* (Joe, 1971).

Figure 7 shows that the perceived benefits obtained by *subordinates outside the circle* with high internal control personality from deviant innovative behavior show an upward trend and the value is positive. Figure 8 shows that the perceived benefits obtained by *subordinates outside the circle* with low internal control personality from deviant innovative behavior show a downward trend and the value tends toward 0, indicating that *subordinates outside the circle* with high internal control personality tend to regard deviant innovative behavior as an opportunity for role change, while *subordinates outside the circle* with low internal control personality do not believe that their own behavior can play a role change.

### The expected supplementary effect of perceived benefits on psychological capital

On the premise that *subordinates inside the circle* and *subordinates outside the circle* form their own psychological capital, employees form perceived benefits to deviant innovative behaviors based on their own personality characteristics, and decide whether to implement deviant innovative behaviors. As shown in Figure 4, the perceived benefits of high-risk-taking *insider subordinates* to deviant innovative behavior will enhance their psychological capital, thus continuously enhancing the possibility of their deviant innovative behavior. As shown in Figure 5, the perceived benefits of low-risk-taking *subordinates inside the circle* to deviant innovative behavior will weaken their psychological capital, and thus weaken their motivation to implement deviant innovative behavior. As shown in Figure 9, the perceived benefits of deviant innovative behaviors of high internal control personality *subordinates outside the circle* will increase their psychological capital, thus continuously enhancing the possibility of their deviant innovative behavior. As shown in Figure 10, the perceived benefits of *subordinates from outside the circle* with low internal control personality on deviant innovative behavior are not conducive to the rebound of psychological capital, thus weakening their motivation for deviant innovative behavior.

These findings not only imply that the triggering mechanism of deviant innovative behavior of different types of employees is not consistent, but also reveal that whether or not perceived benefits can effectively supplement psychological capital is the deep reason why employees make deviant innovative behavior decisions. In other words, *insider subordinates* need high risk-taking traits to activate their deviant innovative behaviors, and *outsider subordinates* need both the desire for level variation and high internal control personality to activate their deviant innovative behaviors.

## Data availability statement

The datasets presented in this article are not readily available because it’s an ongoing study and the data will be confidential until the project is completed. Requests to access the datasets should be directed to MW, mewu@ujs.edu.cn.

## Author contributions

All authors listed have made a substantial, direct, and intellectual contribution to the work, and approved it for publication.
